# Dual Pathology in Rasmussen's Encephalitis: A Report of Coexistent Focal Cortical Dysplasia and Review of the Literature

**DOI:** 10.1155/2012/569170

**Published:** 2012-09-29

**Authors:** Richard A. Prayson

**Affiliations:** Department of Anatomic Pathology, Cleveland Clinic, 9500 Euclid Avenue, Cleveland, OH 44195, USA

## Abstract

Rasmussen's encephalitis is a well-established, albeit rare cause of medically intractable epilepsy. In a small number of Rasmussen's cases, a second pathology is identified, which independently can cause medically intractable seizures (dual pathology). This paper documents a case of a 13-year-old male who presented with medically intractable epilepsy. The patient underwent a series of surgical resections, early on resulting in a diagnosis of focal cortical dysplasia and later yielding a diagnosis of coexistent Rasmussen's encephalitis, marked by chronic inflammation, microglial nodules, and focal cortical atrophy, combined with focal cortical dysplasia (Palmini et al. type IIA, ILAE type IIA). The literature on dual pathology in the setting of Rasmussen's encephalitis is reviewed.

## 1. Introduction

Rasmussen's encephalitis is a rare, progressive disorder of childhood associated with hemispheric atrophy, intellectual decline, and hemiparesis [1–3]. It is a well-established cause of medically intractable seizures, often necessitating surgical resection after failed attempts at pharmacologic management of the seizures [4–6]. Although the pathologic findings often resemble that of a viral encephalitis, attempts at identifying a viral etiology have been mixed and reliable identification of an offending infectious agent has not been successful. Given the presence of autoantibodies in many cases, particularly GluR3 autoantibodies, a variety of immunotherapy treatments have been attempted with varied success [7–9].

In large series studying tissues resected in the setting of medically intractable epilepsy, a variety of other pathologies have been more commonly identified. In most series, the most commonly identified pathologies include mesial temporal or hippocampal sclerosis (in patients with temporal lobe epilepsy), focal cortical dysplasia, tumors, and remote infarcts/ischemic damage [10–13]. In a subset of these cases, dual pathology or coexistent pathologies has been noted—patients in whom two or more pathologies are identified in the histopathological examination of resected tissues, each of which could independently account for the seizures. Only rare cases of dual pathology involving Rasmussen's encephalitis as one of the pathologies have been reported [14–19].

The current paper documents an unusual case which was initially diagnosed as focal cortical dysplasia; the patient underwent a series of seven surgical procedures for intractable epilepsy before a diagnosis of concomitant Rasmussen's encephalitis was made. The literature documenting dual pathology in the setting of Rasmussen's encephalitis is reviewed.

## 2. Case Report

The patient is a 13-year-old male who initially presented at the age of 4 years with seizures, marked by staring, twitching of the left eyelid and projectile vomiting. He experienced anywhere between 10–60 seizures a day. Starting at the age of 7 years, after multiple failed attempts to control the seizures using various pharmacologic regimens, the patient underwent the first of 7 surgical resections. The first two surgeries were performed outside the United States and consisted of resections of the left hippocampus and neocortex and the left anterior temporal lobe and hippocampus. Nonspecific diagnoses of gliosis were reportedly made in examining the resected tissue from the first two surgeries. A third surgical resection for continued seizures at the age of 10 years included resections of the left frontal lobe and portion of the left temporal lobe; the pathologic diagnosis was focal cortical dysplasia, Palmini type IA [19]. The patient was seizure-free for 14 months postoperatively but eventually developed seizure recurrence. Three subsequent surgeries within a 3-week period ensued and included resections of the left temporal lobe, orbitofrontal region, and insula. The pathology in all three cases was interpreted as representing Palmini type IA focal cortical dysplasia. Seizures recurred within 2 weeks of the sixth surgery and persisted for the next 5 months. A seventh surgery included a left frontoparietal resection.

**Figure 1:**
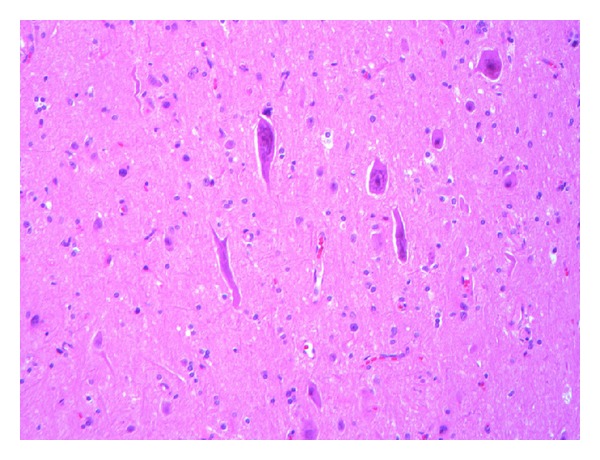
A focus of enlarged, dysmorphic neurons is located in cortical layer 3 from the left frontal lobe, consistent with a Palmini et al. type IIA focal cortical dysplasia (hematoxylin and eosin, original magnification ×200).

**Figure 2:**
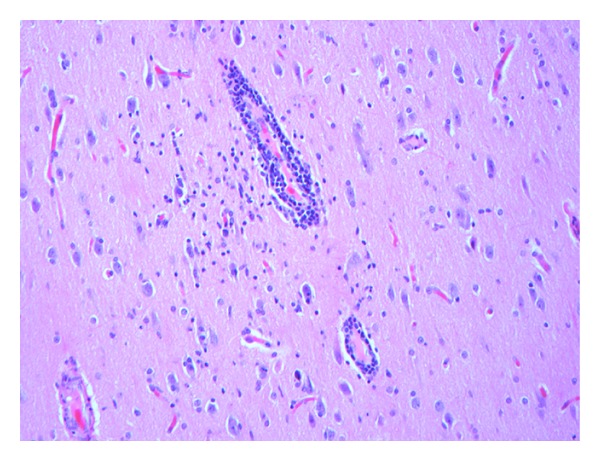
Perivascular chronic inflammation involving cortical blood vessels with an adjacent microglial cell proliferation is present. Meningeal perivascular chronic inflammation is also observed (not seen here) (hematoxylin and eosin, original magnification ×200).

**Figure 3:**
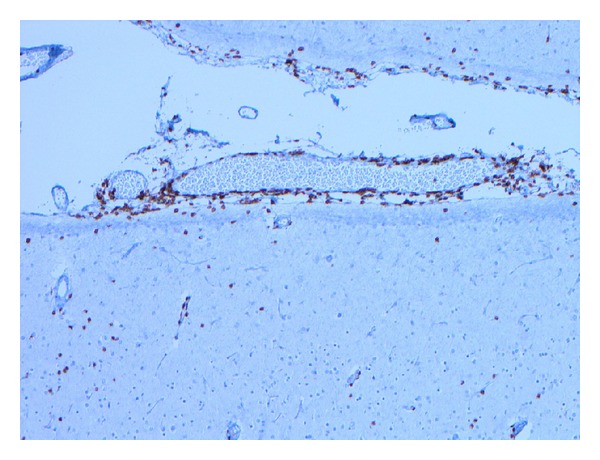
Perivascular meningeal lymphocytes and scattered parenchymal lymphocytes demonstrate positive staining with antibody to CD3 (T-cell lymphoid marker). The vast majority of lymphocytes seen were T cells (CD3 immunostain, original magnification ×100).

**Figure 4:**
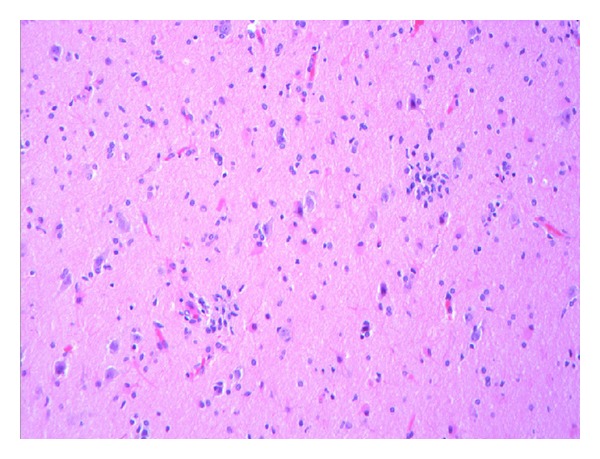
Two cortical microglial nodules are present in this field. Viral inclusions were not seen in association with the nodules (hematoxylin and eosin, original magnification ×200).

**Figure 5:**
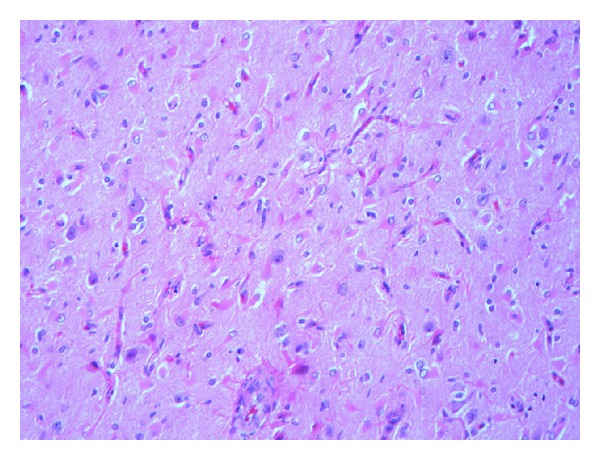
A loss of cortical neurons accompanied by prominent reactive astrocytosis marks an area of cortical atrophy (hematoxylin and eosin, original magnification ×200).

Histopathologic examination of tissue from the final surgical resection showed focal evidence of contusional damage, related to previous surgery. Areas of cortical architectural disorganization marked by an abnormal layering pattern with focal absence of cortical layer two and occasional enlarged and dysmorphic neurons characterized by an atypical distribution of Nissl substance in the cytoplasm were observed (Figure 1). The findings were interpreted as being consistent with a Palmini type IIA pattern of focal cortical dysplasia. Balloon cells were not identified. Additionally, multiple foci of perivascular meningeal and parenchymal chronic inflammation consisting of benign appearing lymphocytes were observed (Figure 2). Most of the lymphocytes stained with T-cell antibody to CD3 (prediluted, Ventana, Tucson, AZ, USA) (Figure 3); only rare CD20 (1 : 25 dilution, Dako, Carpenteria, CA, USA) positive staining B lymphocytes were present. Infiltration of the parenchyma by scattered lymphocytes was highlighted on the CD3 immunostain. Distributed primarily within the cortex were small collections of microglial nodules, highlighted with an CD68 immunostain (1 : 60 dilution, Dako, Carpenteria, CA, USA) (Figure 4). Viral inclusions were not identified. Neutrophils, eosinophils and granulomatous inflammation were not observed. Focally, prominent cortical atrophy with gliosis was observed (Figure 5). The histopathologic findings were interpreted as representing Rasmussen's encephalitis associated with focal cortical dysplasia (Palmini et al. type IIA pattern or ILAE focal cortical dysplasia type IIId) [20].

## 3. Discussion

In 1958, Rasmussen et al. described three patients who suffered from seizures due to chronic localized encephalitis [1]. Subsequently, Rasmussen's name has become associated with this generally unilateral, hemispheric condition. Pathologically, the salient features include chronic meningeal and parenchymal inflammation, consisting primarily of benign appearing T lymphocytes, both diffuse and nodular microglial cell proliferation, gliosis, and neuronal cell death [3, 6, 21]. The underlying pathogenesis of the disorder is still of some debate. Evidence supporting GluR3 autoantibody-induced injury is conflicting and there are cases in which such autoantibodies do not appear to exist; however, there is evidence to support a T-cell-mediated cytotoxicity targeting neurons in the disorder [6]. Treatment strategies have employed various combinations of surgery and immunotherapy, with the goals of decreasing seizure frequency and slowing or preventing further neurologic deficits from developing; treatment results have been mixed.

In rare cases, a second pathology is identified in the background of Rasmussen's encephalitis which independently could cause chronic epilepsy. In 1996, Yacubian et al. described a case of Rasmussen's encephalitis presenting in a 7-year-old girl with epilepsia partialis continua [14]. Resected tissue additionally showed evidence of cortical layer blurring with clusters of dysplastic neurons in the cortex, marked by coarse, maloriented dendrites; balloon cells were not identified. Although predating the development of the Palmini et al. classification, the findings appear to be consistent with the Palmini et al. type IIA focal cortical dysplasia (or ILAE type IIA focal cortical dysplasia).

Two years later, Hart and colleagues reported five cases of so-called dual pathology in Rasmussen's syndrome [16], indicating a 10% incidence of such findings in their series of patients. Among the secondary pathologies described in their patients were two cases of coexistent vascular abnormalities resembling cavernous angiomas, one tumor (possible juvenile pilocytic astrocytoma), one case of tuberous sclerosis and one forme fruste of tuberous sclerosis. Subsequent case reports of concomitant cortical dysplasia (by description corresponding to Palmini et al. type I focal cortical dysplasia or ILAE type Ic focal cortical dysplasia) [18], ganglioglioma [17] and “type II” cortical dysplasia with old ischemic changes were published [15].

Most recently, Takei et al. reported a small series of seven pediatric patients with dual pathology Rasmussen's encephalitis/focal cortical dysplasia, one of whom also had Ammon's horn sclerosis [13]. The authors indicated that they did not classify the cortical dysplasia since most of their cases seemed to have overlapping features of Palmini et al. type I and II lesions with the notable absence of balloon cells in all cases. By description, three cases likely had Palmini et al. type IA or ILAE type Ic lesions and the remaining four cases contained “large” (Palmini et al. IB) “and/or misshapen dysplastic immature-appearing neurons” (Palmini et al. type IIA or ILAE type IIa lesions).

Collectively in reviewing the literature, it would appear that all cases involved pediatric patients with age of seizure onset in the first decade of life, similar to the classic nondual pathology cases of Rasmussen's encephalitis. In all cases, the preoperative imaging studies did not suggest dual pathology, although a differential of Rasmussen's encephalitis with cortical dysplasia was considered in a few cases, based on imaging findings. The current case reported in this study fits this profile. The current case is unique in that the patient underwent multiple surgical resections with an early diagnosis of focal cortical dysplasia being made before tissue diagnostic of Rasmussen's encephalitis was excised.

A few explanations have been offered by way of explaining the presence of dual pathology. One hypothesis suggests that the focal cortical dysplasia lesion and the seizures that might result from it can cause alterations in the blood-brain barrier, allowing circulating antibodies, antigens, and inflammatory cells access to the brain, promoting development of the Rasmussen's encephalitis phenotype [13]. Alternatively, seizures induced by Rasmussen's encephalitis may be responsible for inducing neurogenesis, producing a cortical dysplasia phenotype in the affected area [13].

In the recently published ILAE Task Force classification of focal cortical dysplasia, a new category of type III lesions was added in recognition of the association of focal cortical dysplasia with certain other pathologies [20]. In subtyping these lesions, the current case and other such previously reported cases of dual pathology Rasmussen's encephalitis/focal cortical dysplasia are classified as focal cortical dysplasia type IIId lesions, a somewhat heterogenous group, defined by cortical lamination abnormalities adjacent to other lesions (excepting hippocampal sclerosis, tumor, or vascular malformation) acquired during early life (such as trauma, ischemic injury, or encephalitis). It is unclear at this point whether such a distinction has any clinical implication in terms of seizure outcomes or prognosis.
